# Additional value of first pass magnetic resonance myocardial perfusion imaging to computed tomography coronary angiography for detection of significant coronary artery disease

**DOI:** 10.1186/1532-429X-11-S1-P187

**Published:** 2009-01-28

**Authors:** Jan GJ Groothuis, Aernout M Beek, Stijn L Brinckman, Martijn R Meijerink, Simon C Koestner, Marco JW Götte, Mark BM Hofman, Albert C van Rossum

**Affiliations:** VU Medical Center, Amsterdam, Netherlands

**Keywords:** Coronary Artery Disease, Compute Tomography Coronary Angiography, Invasive Coronary Angiography, Significant Coronary Artery Disease, Suspected Coronary Artery Disease

## Introduction

As computed tomography coronary angiography (CTCA) has a reported excellent negative predictive value for detection of significant coronary artery disease (CAD), it is increasingly used as first line technique in the evaluation of patients with suspected CAD. However, positive predictive value is low and CTCA lacks information about myocardial perfusion. As first pass magnetic resonance myocardial perfusion imaging (MRMPI) can accurately assess myocardial perfusion and does not involve ionizing radiation, it may be a valuable additional technique to CTCA in the evaluation of patients with suspected CAD. Subsequently, their combined use may lower the number of unnecessary, costly invasive coronary angiographies (CAG).

## Purpose

The additional value of MRMPI to CTCA for detection of significant CAD was investigated using invasive coronary angiography as the standard of reference.

## Methods

Patients with chest pain and intermediate pre-test probability CAD underwent both 64-slice CTCA (Sensation, Siemens, Erlangen) and adenosine stress and rest first pass MRMPI (1,5 Tesla MR scanner, Siemens, Erlangen). CTCA was scored per segment as: normal; non-obstructive CAD (0–50% diameter stenosis) and abnormal (>50% stenosis). MRMPI was analyzed qualitatively and was assessed as abnormal in case of any segment with a perfusion defect. In case of abnormal CTCA and/or abnormal MRMPI, CAG was performed. Significant CAD was defined as > 70% diameter stenosis on CAG.

## Results

**Figure 1:**
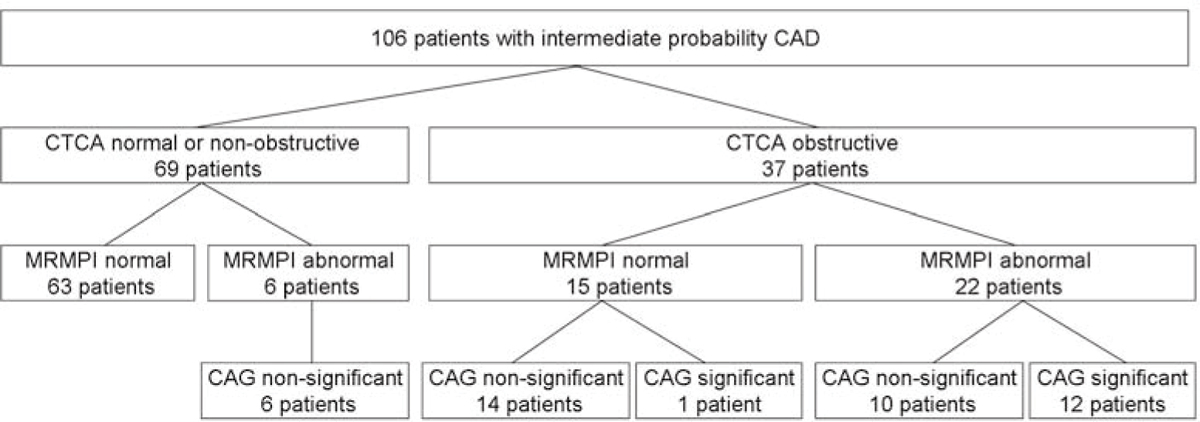
Figure 1

A total of 106 patients (mean age 57 ± 10; 53 males) underwent both CTCA and MRMPI within 2 weeks. 43 Patients underwent CAG. Of 69 patients with normal or non-obstructive CTCA, 6 patients had abnormal MRMPI. None of these patients had significant CAD on CAG. Of 37 patients with abnormal CTCA, 13 patients had significant CAD on CAG (positive predictive value 35%). Of 37 patients with abnormal CTCA, 15 (41%) patients had normal MRMPI. In 14 of these 15 patients absence of significant CAD was confirmed by CAG. Figure 1.

## Conclusion

By using MRMPI as additional technique in case of abnormal CTCA findings, significant CAD could be ruled out in 14 of 37 (38%) patients with abnormal CTCA. In case of normal CTCA however, the additional value of MRMPI for detection of significant CAD is low.

